# V1bR enhances glucose-stimulated insulin secretion by paracrine production of glucagon which activates GLP-1 receptor

**DOI:** 10.1186/s13578-024-01288-4

**Published:** 2024-08-31

**Authors:** Ying Yun, Shimeng Guo, Xin Xie

**Affiliations:** 1 Shandong Laboratory of Yantai Drug Discovery, Bohai Rim Advanced Research Institute for Drug Discovery, Yantai, China; 2 State Key Laboratory of Drug Research, National Center for Drug Screening, Shanghai Institute of Materia Medica, Chinese Academy of Sciences, 189 Guo Shou Jing Road, Shanghai, 201203 China; 3 School of Pharmaceutical Science and Technology, Hangzhou Institute for Advanced Study, University of Chinese Academy of Sciences, Hangzhou, 310024 China

**Keywords:** Arginine vasopressin, Insulin, Glucagon, V1bR, GLP-1R, Islets, Diabetes

## Abstract

**Background:**

Arginine vasopressin (AVP) has been reported to regulate insulin secretion and glucose homeostasis in the body. Previous study has shown that AVP and its receptor V1bR modulate insulin secretion via the hypothalamic-pituitary-adrenal axis. AVP has also been shown to enhance insulin secretion in islets, but the exact mechanism remains unclear.

**Results:**

In our study, we unexpectedly discovered that AVP could only stimulates insulin secretion from islets, but not β cells, and AVP-induced insulin secretion could be blocked by V1bR selective antagonist. Single-cell transcriptome analysis identified that V1bR is only expressed by the α cells. Further studies indicated that activation of the V1bR stimulates the α cells to secrete glucagon, which then promotes glucose-dependent insulin secretion from β cells in a paracrine way by activating GLP-1R but not GCGR on these cells.

**Conclusions:**

Our study revealed a crosstalk between α and β cells initiated by AVP/V1bR and mediated by glucagon/GLP-1R, providing a mechanism to develop new glucose-controlling therapies targeting V1bR.

**Supplementary Information:**

The online version contains supplementary material available at 10.1186/s13578-024-01288-4.

## Introduction

Arginine vasopressin (AVP), also known as antidiuretic hormone, is a neuropeptide composed of nine amino acids [1]. In the 1980s, scientists discovered this small peptide with vasoconstrictive effects in extracts from the posterior pituitary gland, which initiated the studies on AVP and its physiological functions [2]. AVP receptors include three subtypes, namely V1a, V1b and V2 receptors [3]. V1a receptor is predominantly expressed in smooth muscle cells. Activation of V1a receptor induces contraction of vascular smooth muscle [4]. V2 receptor is mainly distributed on the lateral surface of the basal cells of renal tubules near the collecting ducts. The activation of V2 receptor promotes recruitment of aquaporin 2, which increases permeability of the epithelial cell membrane to water and facilitates kidney water reabsorption [5]. V1b receptor is highly expressed in the pancreas and anterior pituitary and serves as the main mediator of AVP’s functions involved in the regulation of endocrine and metabolism of the body [6, 7].

Several studies have demonstrated the involvement of AVP and V1bR in facilitating insulin secretion and maintaining blood glucose homeostasis. AVP regulates the hypothalamic-pituitary-adrenal (HPA) axis, stimulating the release of corticotropin-releasing hormone (CRH) through V1bR activation, thereby promoting CRH-induced insulin secretion [8]. AVP has been shown to enhance insulin secretion in mouse and rat pancreas, and isolated mouse islets [7, 9, 10]. SSR149415, a selective V1b receptor antagonist, significantly attenuated AVP-stimulated insulin secretion from isolated mouse islets, while the antagonists targeting V1a receptor exert minimal influence [7]. AVP has also been reported to confer protection on islet β cells against cytokine-induced apoptosis [10, 11].

AVP has also been reported to stimulate glucagon secretion from the pancreas of mouse and rat, and the α cell line InR1G9 [2, 12, 13]. Studies on AVP-related neurons have demonstrated that AVP acts as a systemic regulator of glucagon secretion under physiological conditions [14, 15]. The brain perceives glucose concentration to induce AVP secretion, which subsequently influences the pancreas to promote glucagon release and elevate blood glucose levels. However, this regulatory mechanism is impaired in individuals with type 1 diabetes [14]. Compared to wild-type mice, V1b receptor knockout mice have reduced insulin and glucagon levels in the plasma [16]. These findings highlight the crucial roles of AVP and its receptor V1bR in regulating blood glucose homeostasis, and reflect the complex and dual functions of AVP in regulating both glucagon and insulin secretion in animals.

In evaluating AVP-mediated insulin secretion, we unexpectedly discovered that AVP could stimulate insulin release from isolated mouse islets, but not β cells. Single cell analysis of the islets revealed that V1bR is expressed in the α cells but not β cells. Further studies suggested that activation of V1bRs in the α cells promotes the release of glucagon, which then induces insulin secretion from β cells by activating GLP-1 receptor.

## Materials and methods

### Animals

All experimental protocols on animals were approved by the Animal Care and Use Committee at Shanghai Institute of Materia Medica, Chinese Academy of Sciences (IACUC number for C57BL/6J mice: 2023-07-XX-382). C57BL/6J mice were purchased from Slac Laboratory Animal (Shanghai, China) and maintained under a 12 h light/dark cycle with normal chow and free access to water.

### Hormone secretion from isolated islets

Islets were isolated from anesthetized male C57BL/6J mice (8 wk old). 1 mg/mL Collagenase P (Roche, #11213873001) solution was injected into the pancreas via the bile duct, and the pancreas was digested at 37 °C for 15 min. The mixture was centrifuged at 800 rpm for 3 min to collect the precipitate, which was then washed three times with HBSS (5.4 mM KCl, 0.3 mM Na_2_HPO_4_, 0.4 mM KH_2_PO_4_, 4.2 mM NaHCO_3_, 1.3 mM CaCl_2_, 0.5 mM MgCl_2_, 0.6 mM MgSO_4_, 137 mM NaCl, 5.6 mM D-glucose, pH 7.4). Then, the precipitate was re-suspended in HBSS and filtered through a 70 μm filter to obtain islets. The detached islets were cultured overnight in RPMI 1640 supplemented with 10% fetal bovine serum (FBS). The islets were transferred to 48-well plates and starved with KRBB (118.5 mM NaCl, 2.54 mM CaCl_2_, 1.19 mM KH_2_PO_4_, 4.74 mM KCl, 25 mM NaHCO_3_, 1.19 mM MgSO_4_, 10 mM HEPES, pH 7.4) containing 0.5% BSA for 60 min. Subsequently, the islets were incubated with 0.1% dimethyl sulfoxide (DMSO) or compounds for a duration of 2 h at 37 °C in 1 mL KRBB containing 16.8 mM or 2.8 mM glucose. Supernatants were collected, and the insulin concentrations were measured using HTRF Insulin kit (Cisbio, #62INSPEC) and an Envision Plate Reader (PerkinElmer).

### Cell culture and hormone secretion

The INS-1E cells were cultured in RPMI 1640 supplemented with 10% FBS, the αTC1-9 cells were cultured in low glucose DMEM supplemented with 10% FBS and the MIN6 cells were cultured in high glucose DMEM supplemented with 10% FBS. Cells were grown in 96-well plates at a density of 5 × 10^4^ cells (INS-1E and αTC1-9) or 2 × 10^4^ cells (MIN6) per well. After preincubation for 30 min at 37 °C in KRBB, the cells were incubated with either 0.1% DMSO or compounds for 1 h at 37 °C in KRBB containing either 16.8 mM or 2.8 mM glucose. Supernatants were collected and hormone concentrations were measured using HTRF Insulin Detection Kit (Revvity, #62IN1PEH) or HTRF Glucagon Detection Kit (Revvity, #62CGLPEH). For co-culturing of INS-1E and αTC1-9 cells, RPMI 1640 supplemented with 10% FBS was used, both cell densities were 2.5 × 10^4^ cells per well in 96-well plates.

### Calcium assay

V1bR/HEK293 cells were seeded at a density of 4 × 10^4^ cells per well into 96-well culture plates and incubated for 24 h at 37 °C in 5% CO_2_. The cells were then incubated with 2 μM Fluo-4 AM in HBSS at 37 °C for 40 min. After thorough washing, 50 μL of HBSS was added. After addition of 25 μL antagonists and incubation for 10 min at room temperature, 25 μL agonists was dispensed into the well using a FlexStation III microplate reader (Molecular Devices), and the intracellular calcium change was recorded at an excitation wavelength of 485 nm and an emission wavelength of 525 nm.

### cAMP accumulation assay

The cAMP assay was performed with GCGR/HEK293 or GLP-1R/HEK293 cell lines. Briefly, cells were harvested and resuspended in DMEM containing 500 μM IBMX at a density of 2 × 10^5^ cells/mL. Cells were then plated onto 384-well assay plates at 1000 cells/5 μL/well. DMEM (5 μL) containing different concentrations of antagonists were added to the cells and the incubation lasted for 15 min at 37 °C (this step was omitted in the agonist detection), then another 5 μL DMEM containing different concentrations of agonists were added to the cells and the incubation lasted for 30 min at 37 °C. Intracellular cAMP levels were detected with a LANCE Ultra cAMP kit (PerkinElmer, #TRF0264) and an Envision Plate Reader (PerkinElmer) according to the manufacturer’s instructions.

### RNA sequencing of single mouse pancreatic islet cells

Pancreatic islets of male C57BL/6J mice were isolated as previously described and dispersed into single-cell suspension using nonenzymatic Cell Dissociation Solution (Sigma-Aldrich, #C5914) for 3 min at 37 °C. Single islet cells in RPMI 1640 medium (300 cells/μL) were mixed (3:2) with C1 Cell Suspension Reagent before loading onto C1 Integrated Fluidic Circuit (IFC). 20 μL LIVE/DEAD staining solution (2.5 μL ethidium homodimer-1 and 0.625 μL calcein AM in 1.25 mL C1 Cell Wash Buffer) was loaded onto the C1 IFC. Each capture site was carefully examined under microscope in bright field, GFP, and Texas Red channels for cell doublets and viability. Cell lysing, reverse transcription, and cDNA amplification were performed on the C1 Single-Cell Auto Prep IFC. Single cell cDNAs were sequenced by Berry Genomics. The sequencing data were standardized using the Z-score model, cluster analysis and t-SNE analysis were conducted.

### Fluorescence-activated cell sorting and real-time qPCR assay

Pancreatic islets of male MIP-GFP mice (The Jackson Lab, #006864) were isolated and dispersed as previously described. FACS sorting was performed using an Influx cell sorter (BD Biosciences). Forward scatter (FSC) with parallel polarization and side scatter (SSC) were collected at 488 nm with each sample collected directly into Trizol to ensure immediate cell lysis and preservation of RNA integrity. RNA was isolated with guanidine thiocyanate and phenol method and RNA samples were reverse-transcribed using PrimeScript RT reagent Kit (TAKARA, #RR047Q). qPCR was conducted with Hieff qPCR SYBR Green Master Mix (Yeasen, #11202ES). The primer sequences used in qPCR are listed in Table 1.

**Table 1:** Summary of primer and shRNA sequences

Name	Nucleotide sequence
qPCR-mouse *Gapdh*	F: AGGTCGGTGTGAACGGATTTG
	R: TGTAGACCATGTAGTTGAGGTCA
qPCR-mouse *Avpr1b*	F: GAGCCTTCTTGGACTGCTACC
	R: TACAGCCAGGTTGCCTCCT
qPCR-mouse *Ins2*	F: GCTTCTTCTACACACCCATGTC
	R: AGCACTGATCTACAATGCCAC
qPCR-mouse *Gcg*	F: TTACTTTGTGGCTGGATTGCTT
	R: AGTGGCGTTTGTCTTCATTCA
qPCR-mouse *Glp1r*	F: ACGGTGTCCCTCTCAGAGAC
	R: ATCAAAGGTCCGGTTGCAGAA
qPCR-mouse *Gcgr*	F: TGCACTGCACCCGAAACTAC
	R: CATCGCCAATCTTCTGGCTGT
qPCR-rat *Gapdh*	F: ACAGCAACAGGGTGGTGGAC
	R: TTTGAGGGTGCAGCGAACTT
qPCR-rat *Avpr1b*	F: TCTCCGACTCAGCCTTAACCTCAG
	R: CCGTCCACCTGCTCTAAATCCTTC
qPCR-rat *Glp1r*	F: TCCTTCATCCTCCGAGCACTGTC
	R: GCCCAGAGAGTCCTGATACGAGAG
qPCR-rat *Gcgr*	F: CCCAATGTCAGATGGATGAT
	R: TAGCGTGTCTTGAGCAGCCAATC
shRNA- *Glp1r*	F: GCAGAAATGGAGAGAGTATCG
shRNA-*Gcgr*	R: GCAACAGAACTTTCGACAAGT

### shRNA transfection

Specific sequences of shRNAs (Table 1) targeting GCGR or GLP-1R mRNA were constructed into pLKO.1 puro lentiviral vector (Addgene, #8453). Lentiviral vectors and packaging vectors were transfected into HEK293T cells by FuGENE HD Transfection Reagent (Promega, #E2312) to produce virus particles. INS-1E cells were seeded onto 6-well plates at a density of 5 × 10^5^ cells per well and incubated with viruses and 5 μg/mL polybrene for 48 h. Transfected INS-1E cells were collected for real-time qPCR and co-culture experiments.

### Western blot

MIN6 and αTC1-9 cells were lysed and sonicated in 1 × SDS buffer. Aliquots of proteins were fractionated by 10% SDS-PAGE and transferred to polyvinylidene difluoride membranes. The membranes were blocked with 5% nonfat milk for 30 min at room temperature and then incubated overnight at 4 °C in buffer containing anti-GAPDH (Cell Signaling Technology, #14C10 at 1:5000) or anti-V1bR (Abcam, #104365 at 1:900) antibodies. After through washing, the membranes were incubated with proper secondary antibodies for 1 h at room temperature. Immunostaining was visualized using Signal Fire™ ECL Reagent (Cell Signaling Technology, #6883) and images were taken with a ChemiDocXRS imaging system (Bio-Rad).

### Oral glucose tolerance test and insulin detection

For oral glucose tolerance test (OGTT), mice were fasted overnight and then given either 0.1% DMSO in normal saline (vehicle) or 1 mg/kg AVP and 10 mg/kg Exendin (9–39) (*n* = 8 per treatment group) via intraperitoneal injection. A glucose bolus was delivered (1.5 g/kg orally) 15 min later. Blood was collected from a tail nick at designated time points, and plasma glucose levels were determined with a glucose meter. To detect insulin level, blood was collected from the retro-orbital plexus of mice 15 min after glucose administration (*n* = 4 per treatment group) and detected with an ELISA kit for mouse insulin (Crystal Chem, #90080).

### Statistical analysis

Results are presented as mean ± SEM unless otherwise noted. Statistical significance was calculated using two-tailed Student’s t-test, *P* < 0.05 was considered significant. Statistical analysis was conducted using Graph-Pad Prism 8 (www.graphpad.com).

## Results

### AVP induces insulin secretion from islets but not β cell line

**Fig. 1:**
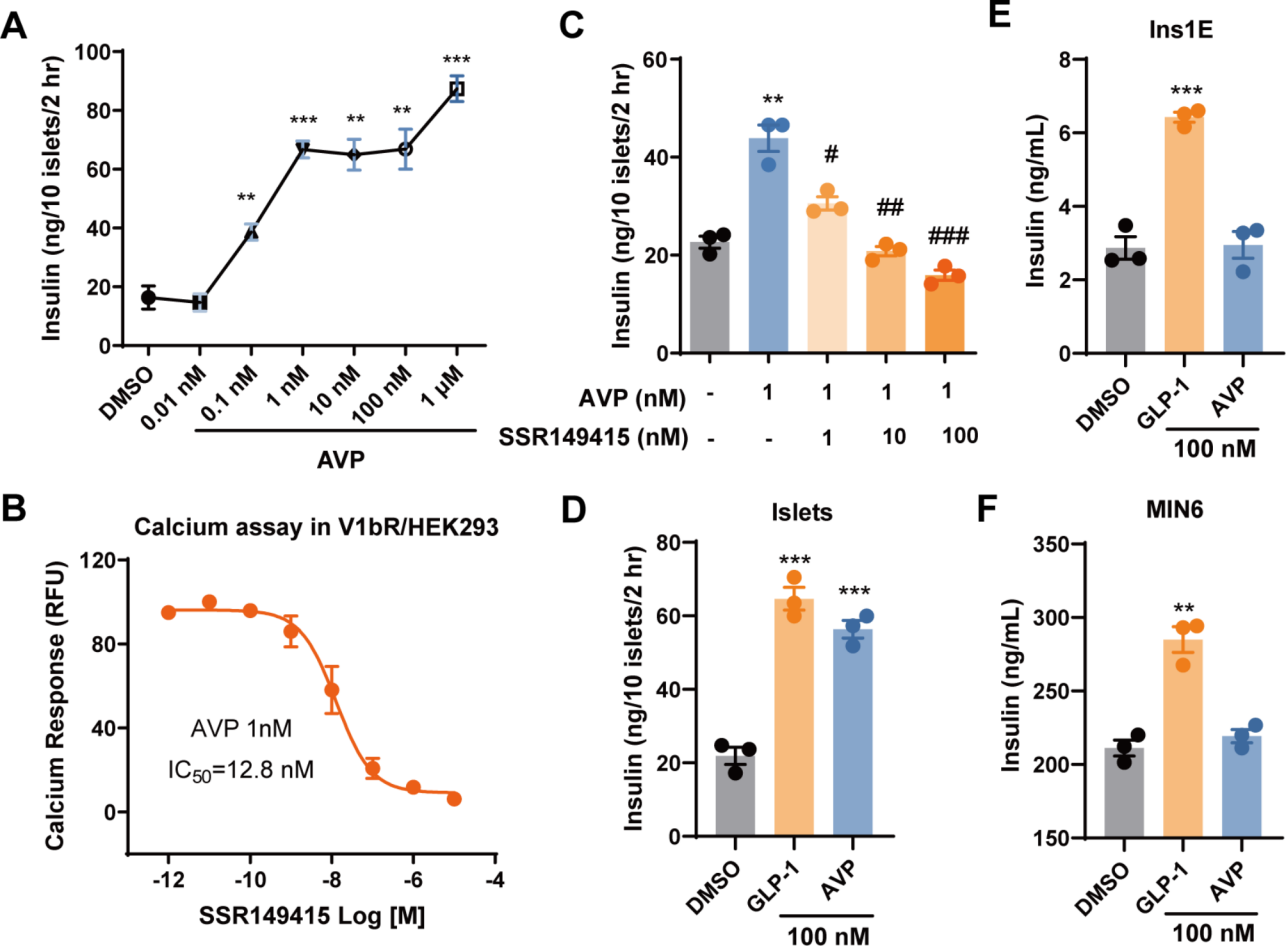
AVP stimulates insulin secretion from mouse islets but not INS-1E cells. (**A**) Insulin secretion from isolated mouse islets (10 islets/well in 48-well plate, incubated in 1 mL KRBB with 16.8 mM glucose) stimulated by various concentrations of AVP for 2 h. (**B**) Dose-response of SSR149415 on AVP (1 nM)-induced calcium signal in HEK293 cells stably expressing V1bR. (**C**) Insulin secretion from isolated mouse islets in the presence of AVP (1 nM) and various concentrations of SSR149415. (**D**) Insulin secretion from isolated mouse islets stimulated by 100 nM GLP-1 or AVP for 2 h. (**E**) Insulin secretion from INS-1E cells (5 × 10^4^ cells/well in 96-well plate, incubated in 100 μL KRBB with 16.8 mM glucose) stimulated by 100 nM GLP-1 or AVP for 1 h. (**F**) Insulin secretion from MIN6 cells (2 × 10^4^ cells/well in 96-well plate, incubated in 100 μL KRBB with 16.8 mM glucose) stimulated by 100 nM GLP-1 or AVP for 1 h. Data are means ± SEM (3 technical replicates). **P < 0.01, ***P < 0.001, versus DMSO control. ^#^*P* < 0.05, ^##^*P* < 0.01, ^###^*P* < 0.001, versus AVP alone

In freshly isolated mouse islets, AVP induced insulin secretion in a dose-dependent manner in high glucose condition (Fig. 1A). SSR149415 [17] is a reported antagonist for V1bR. It dose-dependently inhibited AVP (1 nM)-induced calcium signal in HEK293 cells stably expressing V1bR with an IC_50_ of 12.8 nM (Fig. 1B). SSR149415 also significantly inhibited AVP (1 nM)-induced insulin release from mouse islets at concentrations as low as 1 nM, while higher concentrations of SSR149415 totally abolished AVP-stimulated insulin secretion from the islets (Fig. 1C).

Insulin is exclusively secreted by β cells in the pancreatic islets. To our surprise, although AVP (100 nM) stimulated similar level of glucose-dependent insulin secretion from freshly isolated islets as GLP-1 (Fig. 1D), it failed to induce insulin release from INS-1E (rat insulinoma) or MIN6 (mouse insulinoma), two well-documented cell lines to study insulin secretion and β cell functions, while GLP-1 displayed significant stimulatory effect (Fig. 1E and F). These findings suggest that AVP stimulates insulin secretion from islets in a V1bR dependent manner, but it might not act directly on the β cells.

### V1bR is selectively expressed in the α cells within the islets

**Fig. 2:**
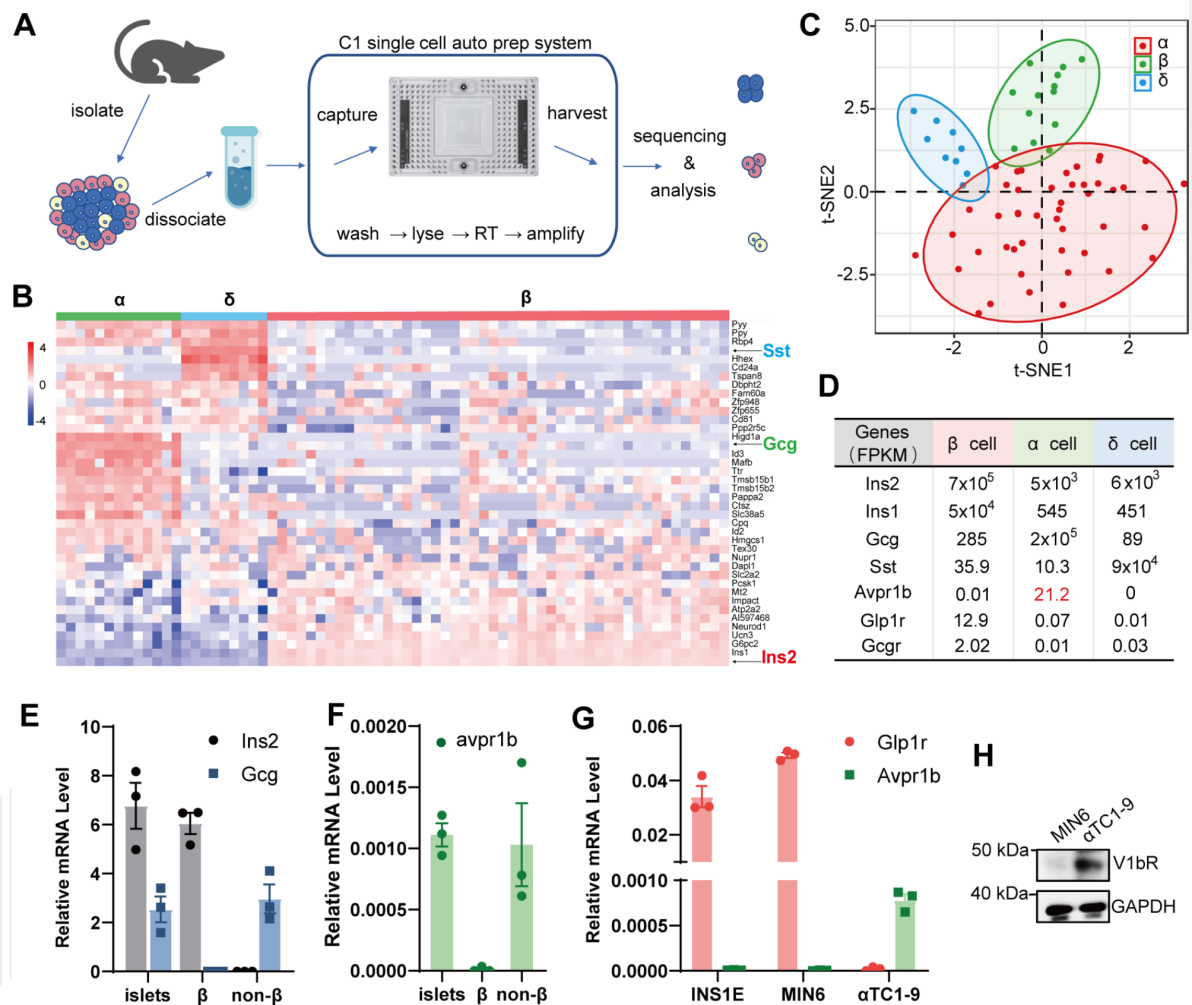
Selective expression of V1bR in α cells of mouse islets. (**A**) Process diagram of single-cell transcriptome sequencing of mouse islets. (**B**) Cluster analysis of the 70 single cells based on differentially expressed genes and well-known marker genes. Gene expression is presented in log 2 scale as defined by the corresponding color bars in the left. Red and blue represent higher and lower gene expression levels respectively. (**C**) tSNE analysis of the 70 single cells using top 50 variable genes. (**D**) Mean FPKM (Fragments Per Kilobase of exon model per Million mapped fragment) of *Ins1*,* Ins2*,* Gcg*,* Sst*,* Avpr1b*,* Glp1r* and *Gcgr* genes in different cell subsets from islet single cell sequencing analysis. (**E**) Relative mRNA level of *Ins2* and *Gcg* in islet β (GFP-positive cells isolated from the islets of MIP-GFP mice in which the GFP expression is controlled by *Ins1* promoter using FACS) or non-β (GFP-negative cells isolated from the islets of MIP-GFP mice using FACS) cells. Data were normalized to *Gapdh* in the same sample. (**F**) Relative mRNA level of *Avpr1b* in islet β or non-β cells. Data were normalized to *Gapdh* in the same sample. (**G**) Relative mRNA level of *Glp1r and Avpr1b* in INS-1E, MIN6 or aTC1-9 cells. Data were normalized to *Gapdh* in the same sample. (**H**) Western blot analysis of V1bR protein levels in the lysates of MIN6 and αTC1-9 cells. GAPDH was used as a loading control. Data are means ± SEM (3 biological replicates)

Islets from C57BL/6 mice were isolated and digested into single cells, and single-cell transcriptome sequencing was utilized to analyze the expression of V1bR (Fig. 2A). The transcriptomes of 70 single cells were extracted, sequenced, and grouped into α, δ and β cells using clustering analysis and tSNE analysis based on significantly differentially expressed genes and related marker genes (Fig. 2B and C). The cells highly expressing *Ins1* and *Ins2* genes were defined as β cells, while the cells highly expressing glucagon (*Gcg*) or somatostatin (*Sst*) genes were defined as α or δ cells (Fig. 2B and D). V1bR (*Avpr1b*) was predominantly expressed in islet α cells with minimal expression observed in islet β or δ cells (Fig. 2D). GLP-1R (*Glp1r*) and GCGR (*Gcgr*) genes could both be detected in β cells, but the expression level of *Glp1r* was much higher (Fig. 2D). To validate these findings, we collected islet β cells and non-β cells using fluorescence-activated cell sorting from the islets of MIP-GFP transgenic mice, which express EGFP under the control of *Ins1* promoter. PCR analysis showed that *Ins2* was only detectable from the β cells and *Gcg* was only observed in non-β cells (Fig. 2E). *Avpr1b* expression could be detected in islets or non-β cells, but not in the β cells (Fig. 2F). *Avpr1b* could also be detected via PCR in αTC1-9 cells (mouse α cell line), but not in INS-1E or MIN6 cells (Fig. 2G). Western blot also confirmed the presence of V1bR protein in αTC1-9 cells but not in MIN6 cells (Fig. 2H)

### Glucagon secreted from α cells by AVP stimulation may facilitate insulin secretion

**Fig. 3:**
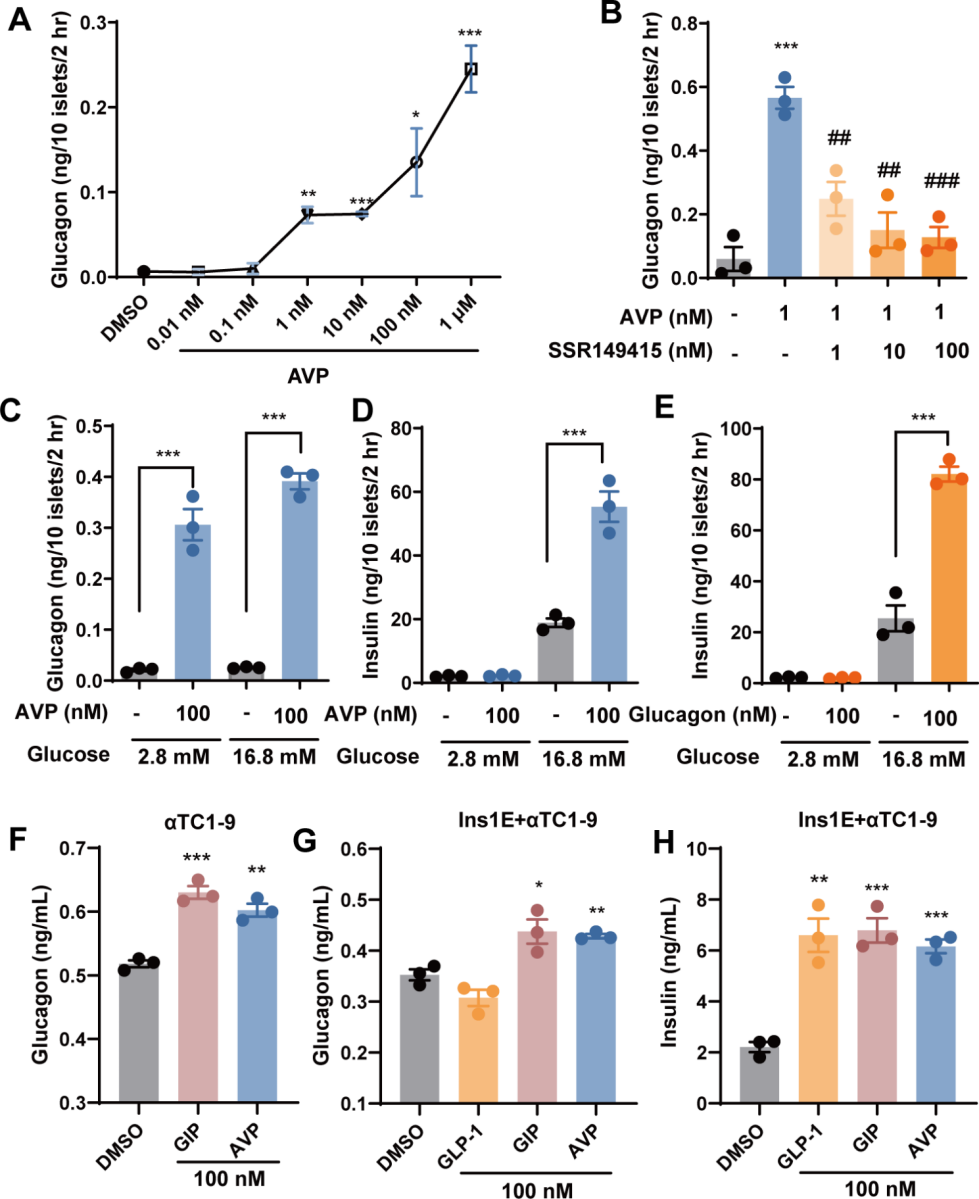
Glucagon secreted from α cells by AVP stimulation may facilitate insulin secretion. (**A**) Glucagon secretion from isolated mouse islets (10 islets/well in 48-well plate, incubated in 1 mL KRBB with 16.8 mM glucose) stimulated by various concentrations of AVP for 2 h. (**B**) Glucagon secretion from isolated mouse islets in the presence of AVP (1 nM) and various concentrations of SSR149415 for 2 h. (**C and D**) Glucagon (C) and insulin (D) secretion from isolated mouse islets stimulated by AVP (100 nM) in both low (2.8 mM) and high (16.8 mM) glucose conditions. (**E**) Insulin secretion from isolated mouse islets stimulated by glucagon (100 nM) in both low (2.8 mM) and high (16.8 mM) glucose condition. (**F**) Glucagon secretion from αTC1-9 cells (5 × 10^4^ cells/well in 96-well plate, incubated in 100 μL KRBB with 16.8 mM glucose) stimulated by 100 nM GIP or AVP for 1 h. (**G and H**) Glucagon (G) and insulin (H) secretion from the co-cultured INS-1E and αTC1-9 cells (both 2.5 × 10^4^ cells/well in 96-well plate, incubated in 100 μL KRBB with 16.8 mM glucose) stimulated by 100 nM GLP-1, GIP or AVP for 1 h. Data are means ± SEM (3 technical replicates). *P < 0.05, **P < 0.01, ***P < 0.001, versus DMSO control. ^##^*P* < 0.01, ^###^*P* < 0.001, versus AVP alone

Since V1bR was only detected in α cells, and α cells are mainly responsible for glucagon secretion, we wondered whether AVP could stimulate glucagon secretion by activating V1bR. Indeed, in isolated mouse islets, AVP induced dose-dependent secretion of glucagon (Fig. 3A), which could be inhibited by SSR149415 in a dose-dependent way (Fig. 3B).

Insulin secretion by pancreatic islets in the body is typically glucose dependent. Islet β cells secrete insulin only in response to high glucose concentrations, while glucagon secretion is not restricted by glucose levels [18]. Indeed, AVP induced glucagon secretion from the isolated islets in low- and high-glucose conditions, while AVP only induced insulin secretion from the islets in high-glucose medium (Fig. 3C and D). This observation aligns with the natural pattern of insulin and glucagon secretions in the body. Adding glucagon to the isolated islets also induced insulin secretion in glucose-dependent manner (Fig. 3E). It led us to hypothesize that AVP might stimulate α cells to release glucagon which in turn induces glucose-dependent insulin release from the β cells.

Since AVP could not directly induce insulin secretion from INS-1E cells, a co-culture of α and β cell lines was carried out. First, we confirmed that AVP, like GIP, could induce glucagon release from αTC1-9 cells (Fig. 3F). Then, in the co-culture of INS-1E and αTC1-9 cells, both AVP and GIP were found to induce secretion of glucagon and insulin in high-glucose condition, while GLP-1 only induced insulin secretion (Fig. 3G and H). These data clearly indicate that the insulinotropic effect of AVP is mediated through a paracrine pathway involving glucagon produced by α-cells.

### Glucagon induces insulin secretion mainly by activating GLP-1R on β cells

**Fig. 4:**
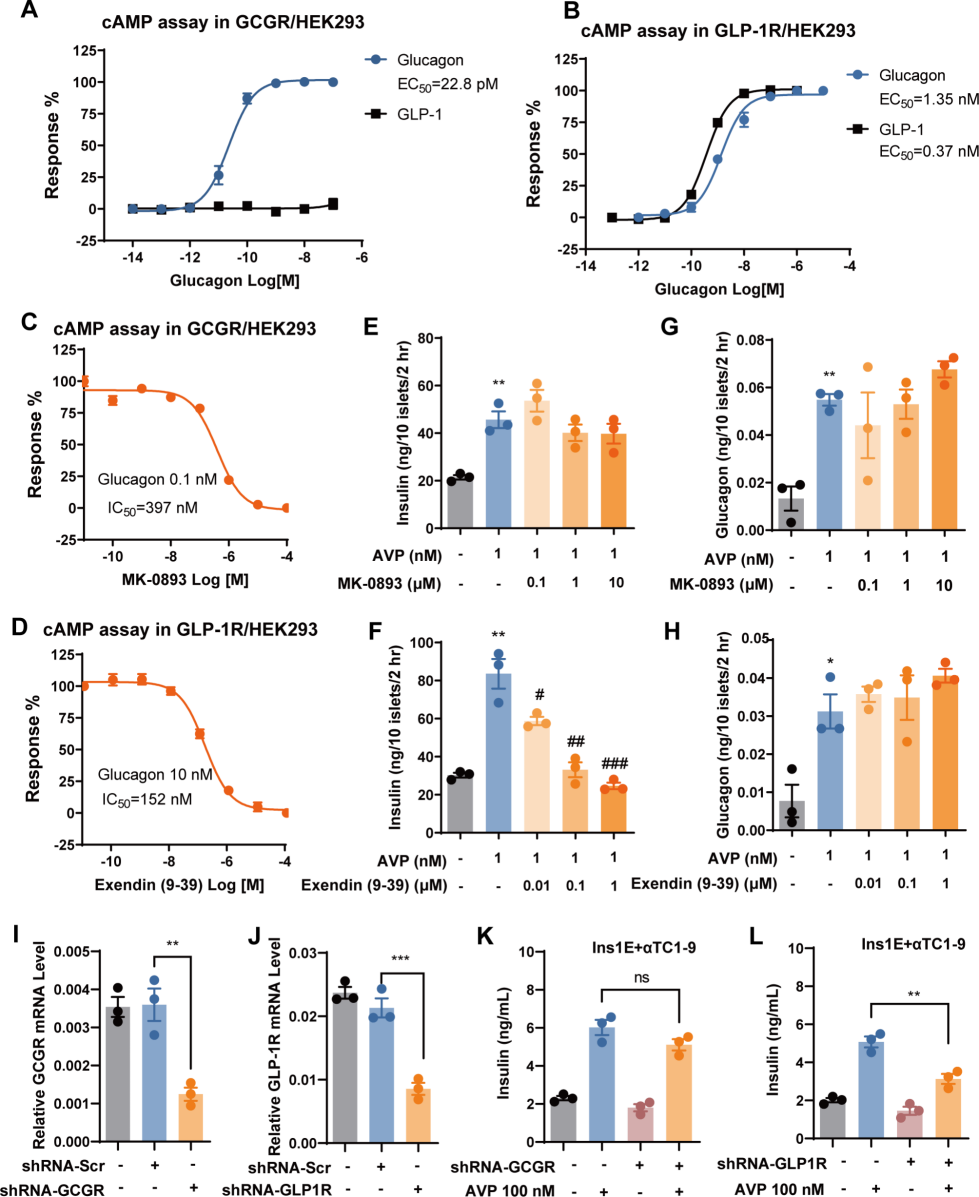
Glucagon stimulated by AVP induces insulin secretion via GLP-1R. (**A** and **B**) Dose-response curves of GLP-1 and glucagon in HEK293 cells stably expressing GCGR (A) or GLP-1R (B). (**C**) Dose-response curves of MK-0893 on glucagon (0.1 nM)-induced cAMP signal in HEK293 cells stably expressing GCGR. (**D**) Dose-response curves of Exendin (9–39) on glucagon (10 nM)-induced cAMP signal in HEK293 cells stably expressing GLP-1R. (**E-H**) Insulin (E and F) and glucagon (G and H) secretion from isolated mouse islets (10 islets/well in 48-well plate, incubated in 1 mL KRBB with 16.8 mM glucose) in the presence of AVP (1 nM) and various concentrations of MK-0893 or Exendin (9–39) for 2 h. (**I** and **J**) Relative mRNA level of *Gcgr* (I) or *Glp1r* (J) in INS-1E cells infected with lentivirus carrying shRNAs targeting *Gcgr* or *Glp1r*, scrambled shRNA (shRNA-Scr) was used as control. Data were normalized to *Gapdh* in the same sample. (**K** and **L**) Insulin secretion levels from co-culture of INS-1E (infected with shRNA-*Gcgr* (K) or shRNA-*Glp1r* (L)) and αTC1-9 cells (both 2.5 × 10^4^ /well in 96-well plate, incubated in 100 μL KRBB with 16.8 mM glucose) stimulated by AVP (1 nM) for 1 h. Data are means ± SEM (3 technical replicates). *P < 0.05, **P < 0.01, ***P < 0.001, versus DMSO control. ^#^*P* < 0.05, ^##^*P* < 0.01, ^###^*P* < 0.001, versus AVP alone

Both GCGR and GLP-1R have been detected in islet β cells [19, 20], which was also observed in our study, although the expression level of *Gcgr* was much lower than *Glp1r* (Fig. 2D). Both receptors can be activated by glucagon [21]. Our data confirmed that glucagon could indeed activate both GCGR and GLP-1R with EC_50_s of 22.8 pM and 1.35 nM, respectively, while GLP-1 could only activate GLP-1R (Fig. 4A and B). Therefore, AVP-stimulated glucagon from α cells may activate one or both receptors to stimulate insulin secretion from β cells.

MK-0893, a specific antagonist of GCGR [22], and Exendin (9–39), a specific antagonist of GLP-1R [23], were validated to show potent and dose-dependent antagonistic effect on GCGR and GLP-1R, respectively (Fig. 4C and D). MK-0893 did not affect AVP-induced insulin secretion from isolated islets at concentrations up to 10 μM, while Exendin (9–39) almost totally abolished AVP-stimulated insulin secretion from islets at 0.1 μM (Fig. 4E and F). Both antagonists did not affect AVP-induced glucagon secretion from the islets (Fig. 4G and H). Furthermore, knocking down *Glp1r* in INS-1E cells using shRNA significantly attenuated AVP-induced insulin secretion from the co-culture of INS-1E and αTC1-9 cells, while shRNA targeting *Gcgr* had no significant effect (Fig. 4K and L). It is noteworthy that the qPCR analysis further confirmed that the expression of *Gcgr* is much lower than *Glp1r* in β cell line (Fig. 4I and J), consistent with our single cell analysis of the islets (Fig. 2D). This may explain the lack of effects upon GCGR antagonist administration or knockdown of *Gcgr* expression.

### GLP-1R antagonist inhibits AVP-improved glucose tolerance in mice

**Fig. 5:**
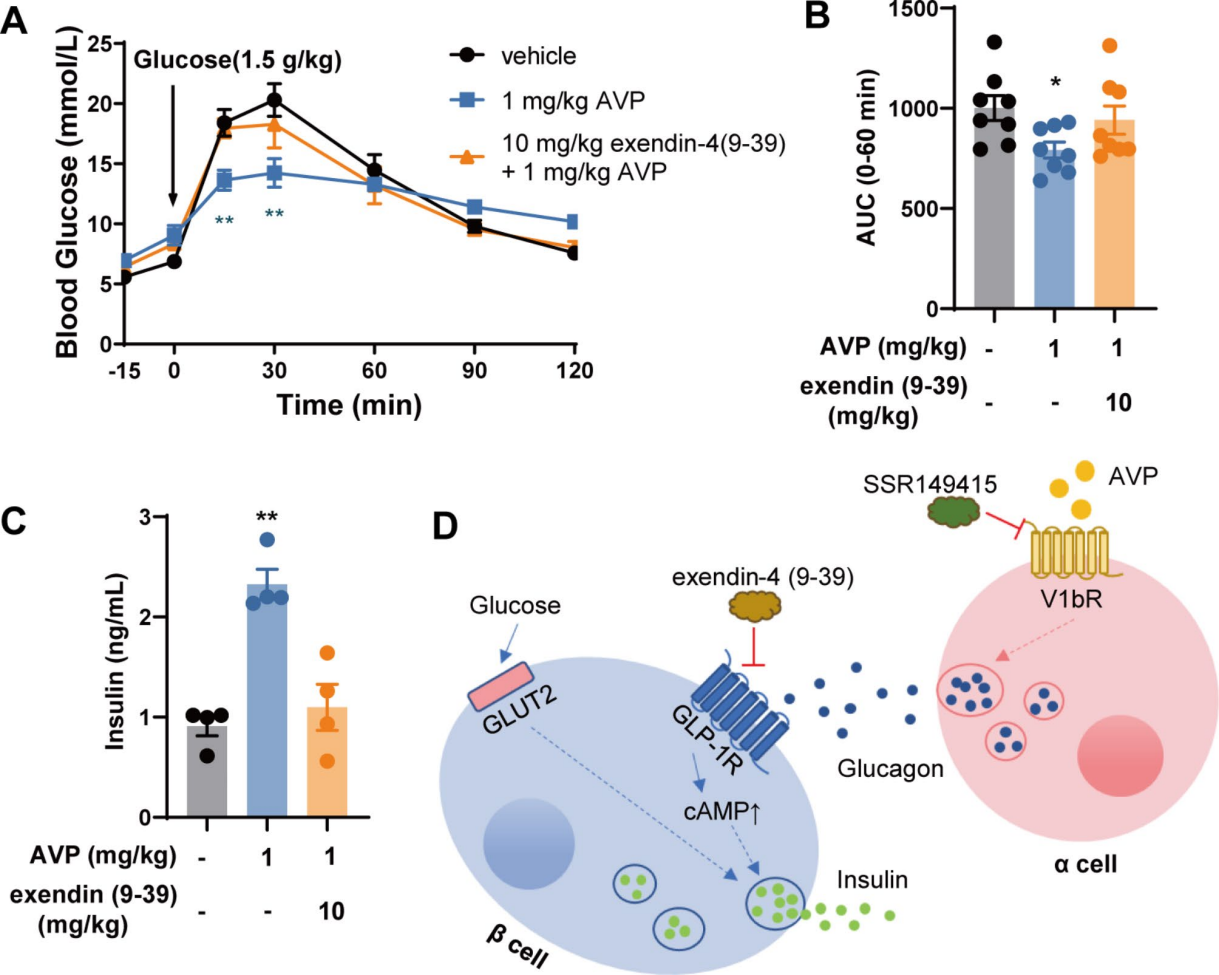
Inhibition of AVP-improved glucose tolerance in mice by GLP-1R antagonist. (**A**) Time-dependent changes of glucose levels in OGTT. C57BL/6J mice were administered AVP (1 mg/kg) and Exendin (9–39) (10 mg/kg) intraperitoneally 15 min prior to a 1.5 g/kg glucose challenge. (**B**) Area under the curve (AUC 0–60 min) of plasma glucose in OGTT presented in (A). Data are means ± SEM (8 mice/group). (**C**) Plasma was obtained 15 min after oral glucose administration in OGTT to measure the levels of insulin. Data are means ± SEM (4 mice/group). *P < 0.05, **P < 0.01, ***P < 0.001, versus vehicle group. (**D**) Schematic representation of the mechanism by which AVP enhances insulin secretion

To validate our findings obtained from the isolated islets and cell lines, we conducted oral glucose tolerance test (OGTT) using 8-week-old C57BL/6J mice. AVP (1 mg/kg) was injected via i.p. 15 min prior to a 1.5 g/kg glucose challenge. AVP significantly lowered blood glucose levels during the OGTT, while co-injection of Exendin (9–39) (10 mg/kg) almost abolished the effect of AVP (Fig. 5A and B). Blood collected 15 min after glucose administration was used to analyze the levels of plasma insulin. AVP significantly stimulated the release of insulin, and this effect was also completely blocked by Exendin 9-39 (Fig. 5C).

Taken together, our data demonstrated that AVP promotes glucagon secretion from the α cells by activating V1bR, and the released glucagon stimulates glucose-dependent insulin secretion from the β cells in a paracrine manner by activating GLP-1R on β cells (Fig. 5D).

## Discussion

The involvement of AVP and V1bR in facilitating insulin secretion is a well-documented phenomenon [7, 9], although the exact mechanism remains elusive. Since V1bR has been reported to be expressed in both the pancreas and anterior pituitary, it may stimulate insulin release via central and peripheral pathways. In the central nervous system, AVP neurons are classified into parvocellular and magnocellular subtypes [24, 25]. Parvocellular AVP neurons project to the median eminence, regulating HPA axis activity. AVP not only stimulates CRH release but also enhances ACTH, corticosterone, and catecholamine secretion within the HPA axis [26, 27], thereby participating in metabolic regulation mediated by these hormones. Magnocellular AVP neurons predominantly contribute to the circulating AVP. Previous investigations using various cell lines and pancreatic perfusion models have demonstrated that peripheral AVP directly promotes insulin secretion in pancreatic islets [9, 10, 13]. In this study, we clearly demonstrate that peripheral AVP stimulates α cells to release glucagon, which in turn stimulates glucose-dependent insulin secretion from the β cells in a paracrine pathway.

Glucagon is typically considered as a glucose increasing hormone by stimulating hepatic glucose output. Although glucagon is used to increase blood glucose and prevent hypoglycemia, both animal and clinical studies have suggested that blocking glucagon signaling rarely causes hypoglycemia [28, 29]. A notion has been proposed that the primary physiological function of glucagon may not lie in the correction of hypoglycemia, and significant elevation of the blood glucose level by glucagon is only observed with pharmacological doses of the peptide [28].

Increasing evidences have suggested that glucagon can potentiate glucose-stimulated insulin secretion from β cells by intra-islet paracrine pathway [30]. Both exogenous and endogenous glucagon (e.g. activation of α cells with alanine) significantly enhanced insulin secretion and reduced glycemia levels in WT mice, but not in mice with double knockout of *Gcgr* and *Glp1r* in β cells [31]. Impairments in insulin secretion were also observed in *Gcg* knockout mice or in inducible α cells ablation models [30, 32, 33]. Some evidence also suggested that a subpopulation of β cells might also express *Gcg* gene which was necessary for normal insulin secretion [34]. These findings indicate that the insulinotropic actions of glucagon can counteract its effects on hepatic glucose output, thereby defining a dual regulatory role of glucagon in metabolic regulation [31].

Despite that glucagon can induce cAMP production by binding to both the GCGR and GLP-1R [35, 36], several evidences suggest that paracrine glucagon effect is mediated primarily by activation of GLP-1R in β cells. Islets isolated from mice with *Gcgr* knockout in β cells and littermate controls displayed identical insulin secretion in response to glucagon, but the deletion of *Glp1r* in β cells critically attenuated glucagon-stimulated insulin secretion [31, 37]. Similar results were obtained by using inhibitors [37]. Antagonism of the GLP-1R blocked large part of glucagon-stimulated insulin secretion, while antagonism of the GCGR only contributed when combined with GLP-1R antagonism, suggesting that the major insulinotropic effect of glucagon is achieved via GLP-1R [37].

Although the mRNAs of both *Glp1r* and *Gcgr* could be detected in β cells, we have demonstrated that the expression level of *Glp1r* is 6–10 times higher than *Gcgr*, that’s probably why glucagon stimulates insulin release from β cells via activation of GLP-1R. Recent studies have demonstrated that consumption of high-fat diet in mice results in the upregulation of *Gcgr* expression and downregulation of *Glp1r* expression within the islets [38]. In this case, activation of GCGR promotes glucose-stimulated insulin secretion more than GLP-1R [38], highlighting the increased significance of GCGR during metabolic stress.

The exact mechanism underlying V1bR-mediated glucagon secretion from α cells remains to be elucidated. However, studies have highlighted the crucial role of the calcium ion in glucagon secretion [39]. V1bR is a GPCR coupled to Gαq protein which induces calcium elevation in cells upon stimulation. Therefore, V1bR is likely to induce glucagon secretion in α cells via the Gαq and calcium pathway.

Even though AVP induces insulin secretion, using high concentrations of AVP to treat diabetes is not advisable due to its many receptors and functions. Epidemiological studies have explored the correlation between AVP and diabetes. Copeptin, a peptide derived from AVP precursor, indicates in vivo levels of AVP. Elevated copeptin concentrations are linked to obesity, insulin resistance, and diabetes onset/progression. Diabetic patients exhibit higher copeptin levels [1, 40]. Moreover, as an antidiuretic hormone, increased secretion of AVP occurs when water intake is insufficient to promote water reabsorption and then elevated concentrations of AVP can affect glucose homeostasis [41]. This theory supports the notion that increasing water consumption may benefit diabetic treatment. Direct application of AVP might not benefic diabetes, but our study suggests targeting pancreatic V1bR might be a more specific way, which warrants further investigation.

### Electronic supplementary material

Below is the link to the electronic supplementary material.

Supplementary Material 1

## Data Availability

The datasets used and/or analyzed during the current study are available from the corresponding author on reasonable request.
