# Supplementary figures and images for: V1bR enhances glucose-stimulated insulin secretion by paracrine production of glucagon which activates GLP-1 receptor
Source: Cell Biosci. 2024 Aug 31;14:110. doi: 10.1186/s13578-024-01288-4 (PMC11365140; doi:10.1186/s13578-024-01288-4)

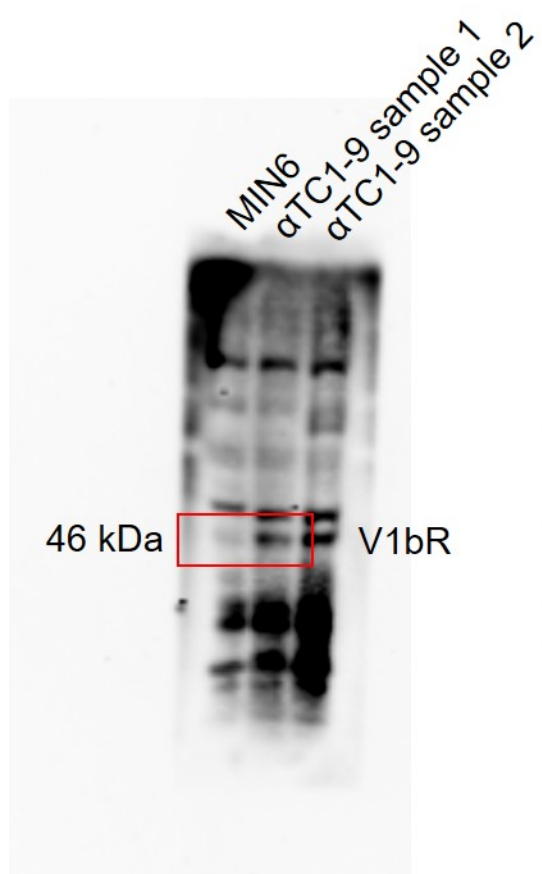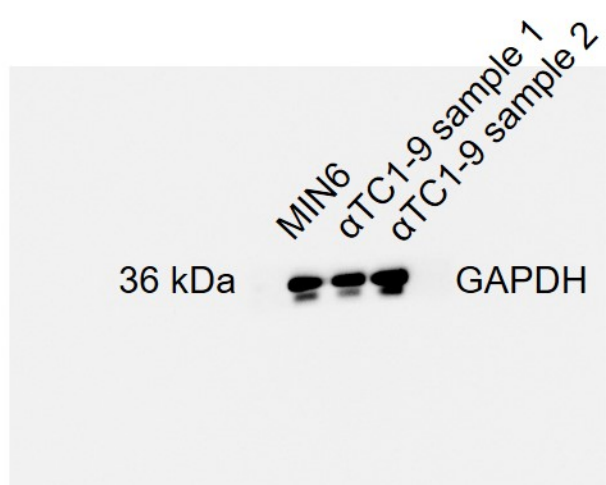

Supplement: Supplementary file 1 — Supplementary Material 1 [file 13578_2024_1288_MOESM1_ESM.pdf]
